# Supplementary material for: Enhancement of short/medium-range order and thermal conductivity in ultrahard sp3 amorphous carbon by C70 precursor
Source: Nat Commun. 2023 Nov 29;14:7860. doi: 10.1038/s41467-023-42195-5 (PMC10686990; doi:10.1038/s41467-023-42195-5)
Supplement: Supplementary file 1 — Supplementary Information [file 41467_2023_42195_MOESM1_ESM.pdf]

# Supplementary Information:

## Enhancement of Short/Medium-Range Order and Thermal Conductivity in Ultrahard $sp^3$ Amorphous Carbon by $C_{70}$ precursor

Shang *et al.*

**Supplementary Note 1. The thermal conductivity measurement of *sp*$^3$ amorphous carbon by using a TDTR method.** The thermal conductivity was measured by using a non-contact time-domain thermoreflectance technique, which is a pump-probe optical method to characterize thermal properties, especially for the samples in micrometer size. The amorphous carbon sample for TDTR measurement is 1 mm in diameter. Before TDTR measurement, the sample was polished and the polished smooth area is about 500 μm in diameter. To avoid the effects of cracks and sample boundary, the laser with a small spot size (spot radius, r=11.6 μm) was used in our thermal conductivity measurement, and TDTR signals only reflect the thermal properties of the laser focus areas. Thus the thermal conductivity properties of amorphous carbon samples can be obtained by measuring the local areas where the sample is dense and crack-free (Supplementary Figs. 4, 5). In order to avoid measurement errors, for each sample, we selected several (at least 3) smooth and crack-free regions randomly for TDTR measurement, and the thermal conductivity value is given by averaging the results of several measurements.

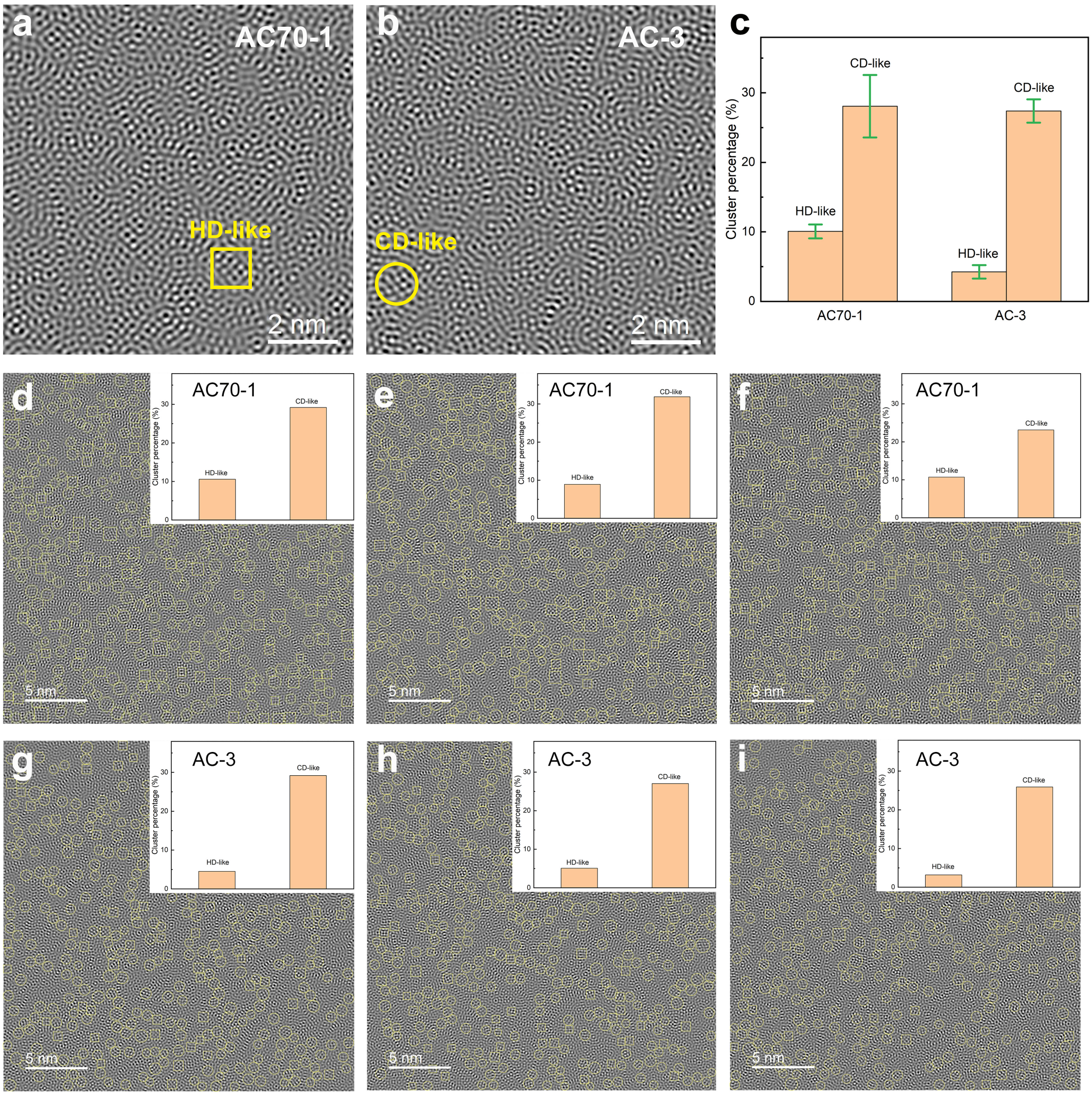


**Supplementary Figure 1. Statistical analysis of the area fraction of MRO clusters for *sp*$^3$ amorphous carbon. a** The typical inverse FFT image of AC70-1, the regions marked with yellow square represent the HD-like MRO cluster. **b** The typical inverse FFT image of AC-3, the regions marked with yellow circle represent the CD-like MRO cluster. **c** Content statistics of diamond-like MRO clusters in AC70-1 and AC-3. Error bars indicate statistical analysis for three different areas, standard deviations. **d-f** Three typical inverse FFT images of AC70-1, the insets are the content statistics of diamond-like MRO clusters in AC70-1. **g-i** Three typical inverse FFT images of AC-3, the insets are the content statistics of diamond-like MRO clusters in AC-3. The regions marked with yellow circles and squares in **d-i** represent the CD-like and HD-like clusters, respectively.

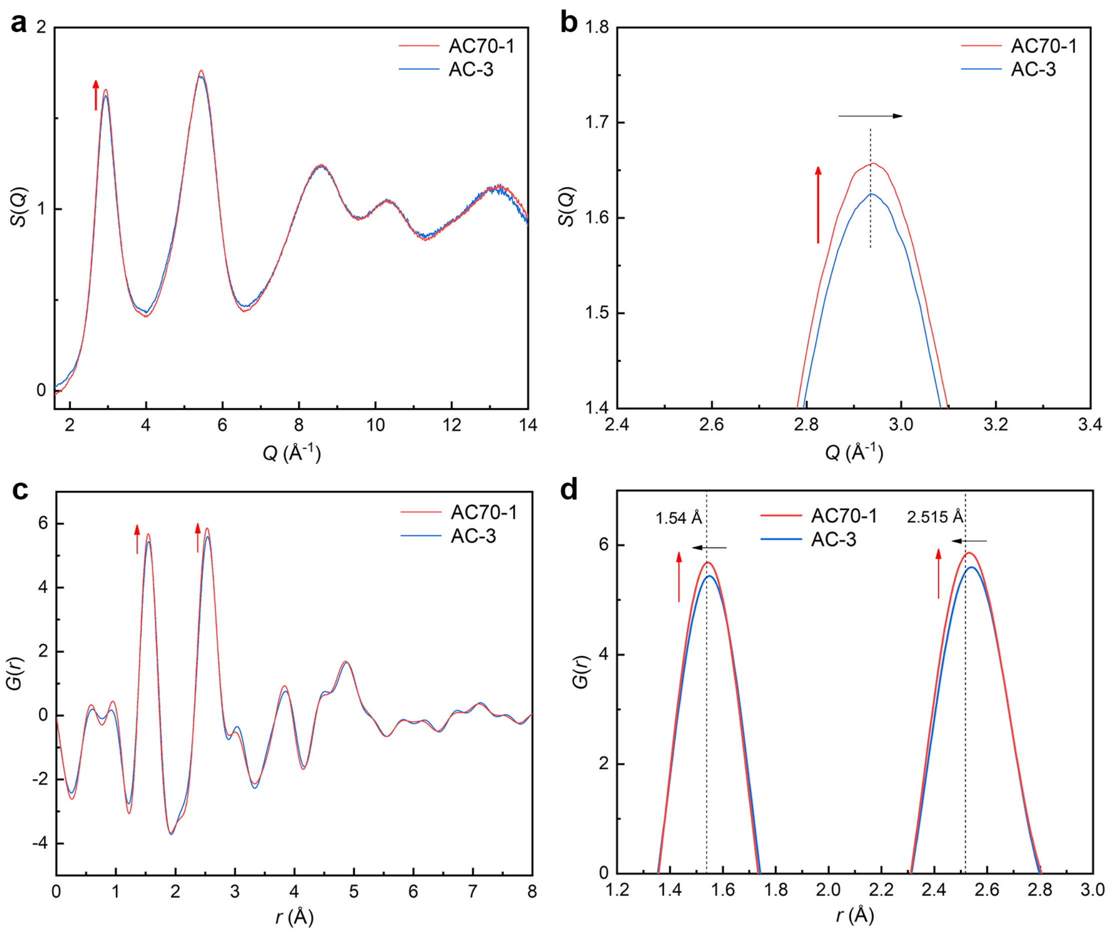


**Supplementary Figure 2. The measured PDF and structure factor data with a $\boldsymbol{Q_{\max}}$ of 14 Å$^{-1}$.** **a** Structure factor $S(Q)$ of AC70-1 and AC-3 samples. **b** The magnified FSDP in $S(Q)$. The dashed line represents the peak position of FSDP for AC-3, which is slightly lower than that of AC70-1. **c** The reduced PDF profiles, $G(r)$, of AC70-1 and AC-3 samples. **d** The magnified first $r_1$ and second $r_2$ peak in $G(r)$. The two dashed lines represent the standard first and second nearest-neighbour atomic distances of crystalline diamond, respectively. The red arrows in **a**, **b**, **c** and **d** indicate the peak intensity increase for AC70-1. The black arrow in **b** indicates the peak position shift to the right for AC70-1. The black arrows in **d** indicate the peak position shift to the left for AC70-1. The diffraction data with a $Q_{\max}$ of 14 Å$^{-1}$ were collected at BL13HB beamline of the SSRF.

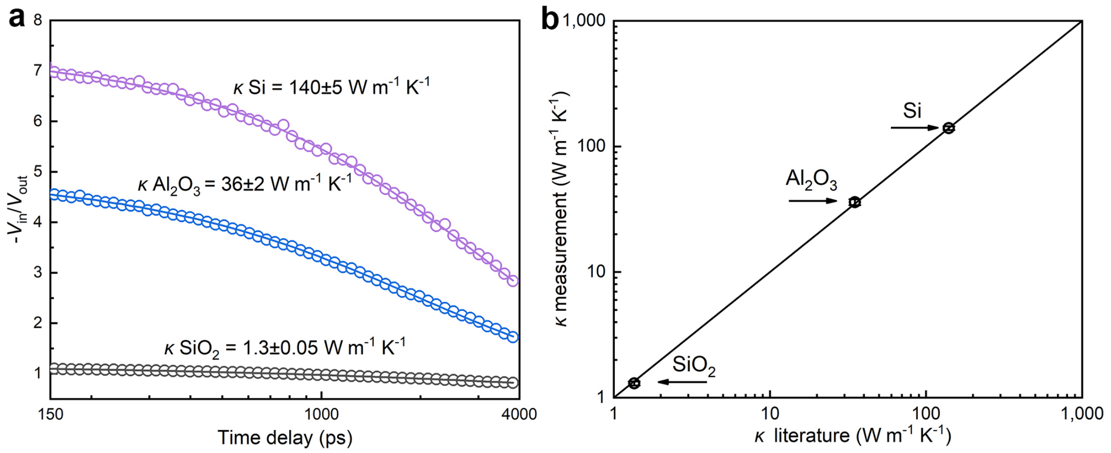


**Supplementary Figure 3. Thermal conductivity measurement of standard samples using the TDTR method. a** The measured TDTR data for the standard Si, $Al_2O_3$ and $SiO_2$ samples. Error bars indicate three different measurement points, standard deviations. **b** The measured thermal conductivities of standard materials compared with literature data.

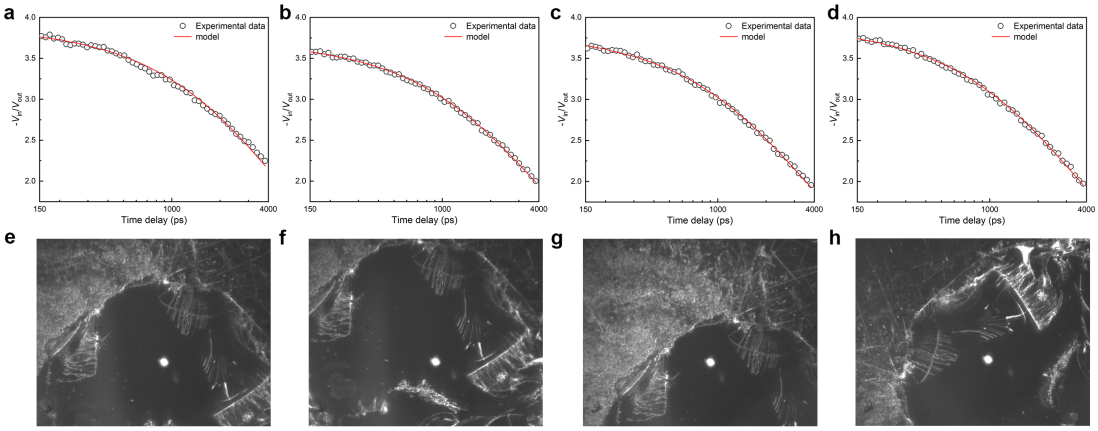


**Supplementary Figure 4. The measured TDTR signals (a-d) and the corresponding four test locations of AC70-1 sample (e-h).** The bright spot in **e-h** is the laser focus position**.** The measured TDTR signals of the four points are almost the same and the results of the four measurements gave an average thermal conductivity of 36.3 W $m^{-1}$ $K^{-1}$ for AC70-1 and a standard deviation of 2.2 W $m^{-1}$ $K^{-1}$.

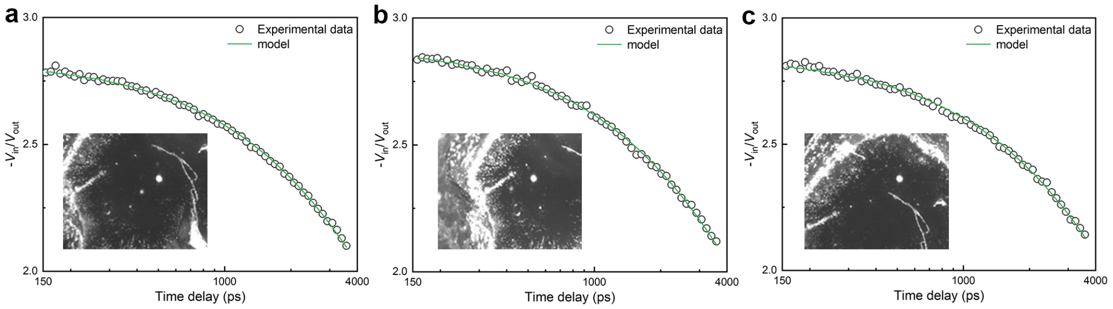


**Supplementary Figure 5. The measured TDTR signals and the corresponding three test locations for AC-3 sample (a-c).** The bright spot is the laser focus position**.** The results of the three measurements gave an average thermal conductivity of 26.0 W $m^{-1}$ $K^{-1}$ for AC-3 and a standard deviation of 1.3 W $m^{-1}$ $K^{-1}$.
